# Expenses of Motor Upkeep

**Published:** 1909-03-27

**Authors:** 


### Motoring Notes.

### EXPENSES OF MOTOR UPKEEP.

The costs incidental to keeping a motor-car are of very great importance to medical men. Many are deterred from purchasing, owing to exaggerated ideas of the cost of running, others on account of the initial outlay, and some because they have a dread of their car going wrong and of lack of confidence in their own ability to diagnose the trouble and cure the disorder. Of the above classes of medical men who hesitate in regard to adoption of the car, I believe the large majority come under the first category. They fully recognise all the advantages of the motor-car, such as extended practice, much shortened duration of their rounds, increased leisure, etc.; but they dread the maintenance expenses, which, perhaps, a few years ago, when the majority of cars were in a more or less experimental stage, were most undoubtedly heavy. At this period the cost of motoring was considerable, and this is remembered whilst few realise the change that, within a few years, has been made in the design and construction of cars and tyres, rendering the motor-car of the present day not only one of the most reliable means of travelling, but also one of the cheapest of all private vehicles to maintain.

This subject of maintenance is of such interest both to those who already use the car and those who are thinking of it that I intend to devote some space to the matter in these columns.

The cost depends on a number of factors, each of which I propose to consider separately.

*Durability of the Car Itself.*—This, of all factors bearing on the expense of car upkeep, is perhaps the most important. It will not suffice if the motor itself is a first-class one. Unless the car is thoroughly reliable and durable in every respect, the cost of maintenance will undoubtedly prove considerable. To buy a car because it is claimed to embody all the latest features and refinements and to be strong and durable is a very grave error. The only real test is the experience of the public after actual use on the road, and to ensure economy it is absolutely necessary to choose a car not only of a make that has been on the market for some considerable time, but one whose details have been thoroughly and severely tested by the general public.

*Number of Miles Travelled.*—It is obvious that other factors being equal, the cost of motoring will depend on the distance covered. This controls everything—consumption of petrol and oil, wear of tyres, etc.—so it must be considered in forming an estimate of the cost of upkeep.

*Weight of the Car when Loaded.*—The heavier the car, the greater the wear on tyres and the higher the consumption of petrol. One should, therefore, in order to lessen the cost of upkeep, select a car not larger than is necessary, and as light as possible without sacrificing the strength and durability of the essential parts.

*Regular and Thorough Lubrication.* — There should, in a first-class car, be few expenses for repairs or replacements if all moving points and surfaces are kept properly lubricated. Thorough lubrication means not only lubrication of the engine, the gear-box, and the road-wheel axles, but also of all other places throughout the chassis where movement occurs. It is remarkable how careful some car owners are to lubricate—indeed, in many instances to over-lubricate—the engine and gears, and yet regularly neglect the less important movements where friction occurs, with the consequent result that they become covered with rust and defective in action. Many cars become old, worn, and noisy before their time, as every repairer knows, simply from want of thorough and systematic attention to this matter, since want of oil and grease causes more damage than any amount of wear, and entails very considerable but unnecessary expenses for re-bushing bearings or renewal of parts.

*The Power and Speed of the Car.*—The higher the power of the engine, the higher will be the consumption of petrol and oil. The higher the power of the engine, the heavier will it not only be itself, but it will necessitate a proportionate increased weight and strength in the chassis, which, as I have pointed out above, also increases the petrol consumption, in addition to causing more wear on tyres. From this point of view it therefore follows that to keep expenses low, a car should be selected of no greater power than is necessary for one's purpose and the nature of the country in which the car will be used.

*Careful Driving.*—Many do not realise the very considerable reduction in the cost of maintenance that can be effected by careful driving. It is well known that a high speed means a much greater proportionate wear on tyres than a moderate one. For this reason alone, not to speak of the danger to oneself and to other road users, high speed should be avoided, and a car should be driven at a moderate rate and with due consideration to the comfort and safety of others. Far too many drivers take corners at too high a speed, let their clutch in harshly, accelerate their engines suddenly, or use their brakes for stopping purposes, instead of slowing down gradually. Such methods of driving, it is apparent, cause greatly increased friction between the tyres and the roads, resulting in very excessive wear of the covers, especially if, as frequently happens, the tyres are insufficiently inflated. The latter condition naturally greatly increases the flexion of the sides and causes rapid disintegration of the covers.

It will be seen from the foregoing that the cost of keeping a motor-car must necessarily depend to a very appreciable extent on two main factors: the first being that a car should be selected that has a record for reliability and durability quite apart from maker's claims, and that it should be of power and weight not beyond what is needed; the second, the attention and care it receives from its owner or driver.

There are one or two other matters in this connection which I propose to touch upon in another issue, especially a very interesting analysis of reports of owners' expenses, compiled by a well-known firm of manufacturers. "Viator."

